# Cirrhosis of the Liver

**Published:** 1904-10-08

**Authors:** 


### Hospital Clinics.

### CIRRHOSIS OF THE LIVER.

A case of cirrhosis of the liver, reported by R. M. McQueen,[1] is not only of interest in itself, but bears upon the relationship existing between the hepatic lesion in cirrhosis and the general symtomatology of the disease. Until a comparatively recent date this relationship was regarded for the most part as a purely mechanical one. The fibrous tissue newly formed in the liver under the influence of alcohol, it was taught, exercised during its contraction pressure on the interlobular branches of the portal vein, and, consequent on this obstruction, there resulted a condition of venous engorgement in the various organs included within the portal circuit. Hence hepatic cirrhosis was marked by such symptoms as gastric catarrh and hæmatemesis, intestinal catarrh with melæna and hemorrhoids, enlargement of the spleen and ascites. Similarly, it was assumed that constriction of the biliary ducts in the liver substance led to the reabsorption of bile with jaundice and poisoning of the cerebral centres (cholæmia). The more modern doctrine teaches that this simple theory of the symptoms associated with cirrhotic changes in the liver is almost certainly incorrect. It suggests, on the other hand, that most, if not all, of the symptoms previously attributed to intra-hepatic venous or biliary obstruction are really results of a toxæmia. The source and nature of the toxic agent are less certain, but it seems not improbable that the poison may arise from the perversion of some internal secretion of the liver. This perversion, it may be assumed, is a direct consequence of the sustained action of alcoholic drinks on the hepatic cells and is not necessarily dependent on the fibrous changes which form the most conspicuous post-mortem record of the disease. Certain it is that the two orders of events, the general symptoms and the hepatic cirrhosis, do not march *pari passu*. And it is by no means unknown to discover, as a matter of fact, an advanced fibrosis of the liver after death though there have been during life none of the symptoms of cirrhosis of the liver in the clinical sense of that term. It is in this particular aspect of the subject that Mr. McQueen's case is of importance. The patient was a man of 56 years and he had enjoyed excellent health until two or three days before his death. He then developed some degree of drowsiness and malaise and in the course of a few hours vomited about a quart of blood. Later there was melæna and the patient became delirious and comatose; the temperature rose to a distinctly febrile level about six hours before death. As an explanation of this rapid and somewhat puzzling combination of symptoms the post-mortem examination revealed conspicuous evidences of hepatic cirrhosis. The liver was not below the normal size, indeed the left lobe was moderately enlarged, but it was "hob-nailed" and showed the macroscopic and microscopic changes familiar in cases of cirrhosis. Thus there could be no doubt at all that in the pathological sense cirrhosis had existed for many months. Yet during this time there had been no reason to regard the patient as other than a healthy man. In other words, the structural changes which formerly were held responsible for the symptoms of the disease had existed without producing these symptoms. The man had enjoyed good health in spite of these changes until suddenly, from some cause or other—possibly a drinking bout—there was produced some agent which caused a fatal toxæmia preceded by a free hæmatemesis. The latter symptom at first sight seems more readily to adapt itself to the mechanical rather than to the toxæmic theory, and it is to be regretted that the absence of a complete post-mortem examination left this point in the case obscure. But it may be remembered that hæmorrhages from mucous membranes are not uncommon in various toxic states, as, for example, in some of the severer forms of certain of the specific fevers, and in purpura hæmorrhagica; and in any event the epistaxis and pharyngeal hæmorrhages which are recognised symptoms in the clinical picture of cirrhosis of the liver can hardly be explained as the consequences of mechanical obstruction in the course of the portal circulation. Therefore, if the epistaxis may be due to a non-mechanical cause the same possibility must be allowed in regard to the hæmatemesis. And thus room is provided here also for the application of the toxæmic theory. In short, the case may be claimed as one tending still further to discredit the mechanical view of the relationship between the symptoms and the hepatic changes found in the disease known as cirrhosis of the liver. A useful summary of the arguments supporting the doctrine of toxæmia is contained in a lecture by Dr. Hale White.[2] In this it is pointed out that early jaundice and œdema of the feet (apart from ascites) both occur in cases of cirrhosis of the liver and that they cannot be due to intra-hepatic obstruction. Similarly, the ascites presents features which make this latter explanation improbable. One of these is the suddenness and rapidity with which it develops; another is the absence of this symptom in many instances, when there is undoubted obstruction to the portal circulation. Hence Dr. Hale White favours the view that ascites, as well as the other symptoms of the disease, is due to the presence of toxic matter in the blood, and that something more than mere increase in the fibrous stroma of the liver is needed to produce the clinical entity known as cirrhosis of the liver. At the same time, it has to be recognised that fluid may accumulate in the peritoneum as the result of a peri-hepatitis or other localised form of peritonitis and that this may be a gradual development. Another fact in Mr. McQueen's case bearing upon current controversies in regard to cirrhotic changes in the liver, was the size of the liver on post-mortem examination. As already stated, whilst the right lobe of the organ was normal in this respect, the left was definitely enlarged. As is well known, there are some authorities who hold that atrophic and hypertrophic cirrhosis are two totally distinct and different diseases, and in certain text-books on medicine the symptoms which distinguish them respectively are presented in sharp contrast. As opposed to this is the view that the two conditions are merely different stages of one and the same disease, and that the hypertrophic form, provided the patient lives sufficiently long, will certainly become atrophic. The case now recorded decidedly favours this latter view.

Finally, a note may be made of the temperature in this case. A febrile rise some few hours before death, however difficult to explain, can hardly be quoted as a tendency to pyrexia. But it has a certain interest arising from the fact that in attempting to draw a distinction between atrophic and hypertrophic cirrhosis stress has been laid on the fact that in the latter alone are temperature disturbances to be observed. There is no doubt at all that pyrexia may be present in cirrhosis of the liver, as seen in groups of cases that have been collected by various authorities.[3] But it is urged that these were all of the hypertrophic variety. This is not very probable, and, even allowing such a possibility, there is at least one instance on record[4] in which, in a quite undoubted instance of atrophic cirrhosis, the thermometer at various times registered a febrile temperature. Leaving out of account the exceptional condition known as Hanot's hypertrophic cirrhosis—a disease usually seen in male children and associated with enlargement of the spleen—the tendency of modern opinion is decidedly towards the view that cirrhosis of the liver is one disease, which in its early stages may be characterised by considerable enlargement of the liver and in its later by atrophic changes; also that whilst for the most part the disease is an apyrexial process, irregular attacks of fever are not so very uncommon.

[1] Lancet, August 20, 1904. [2] The Polyclinic, vol. vi. [3] Guy's Hospital Reports, 1883, and Lancet, 1894, vol. i. [4] C. O. Hawthorne, Brit. Med. Jour., March 16, 1901.

